# Poor glycemic control and its associated factors among children with type 1 diabetes mellitus in Harar, eastern Ethiopia: A cross-sectional study

**DOI:** 10.1186/s12902-023-01453-9

**Published:** 2023-09-28

**Authors:** Betelhem Demeke Habteyohans, Betre Shimelis Hailu, Fentahun Meseret, Ahmed Mohammed, Yeshi Berhanu, Ayichew Alemu, Gadissa Tolosa, Mulualem Keneni, Assefa Desalew

**Affiliations:** 1 Department of Pediatrics and Child Health, School of Medicine, College of Health and Medical Sciences, Haramaya University, Harar, Ethiopia; 2 Department of Pediatrics and Child Health Nursing, School of Nursing and Midwifery, College of Health and Medical Sciences, Haramaya University, P.O. Box 235, Harar, Ethiopia

**Keywords:** Glycemic control, Associated factors, Type 1 child and adolescent, Hiwot Fana Compressive Specialized University Hospital, Harar, Jugol General Hospital, Ethiopia

## Abstract

**Background:**

Poor glycemic control increases the risk of acute metabolic derangements and long-term consequences, which are the main causes of morbidity and mortality. Maintaining adequate glycemic control is challenging for children with diabetes, particularly in resource-limited settings. There is a paucity of data on the magnitude of poor glycemic control and its predisposing factors in Ethiopian particularly in this study setting. Hence, we aimed to assess the magnitude of poor glycemic control and its associated factors among children and adolescents with type 1 diabetic mellitus in Jugol and Hiwot Fana Compressive Specialized University Hospitals in Harar, eastern Ethiopia.

**Methods:**

A facility-based cross-sectional study was conducted among 231 children and adolescents with type 1 diabetes mellitus in Jugol and Hiwot Fana Compressive Specialized University Hospitals. Participants were included consecutively in the follow-up clinic from November 15, 2022 to January 15, 2023. Data were collected through an interviewer-administered structured questionnaire and a review of medical records. A binary logistic regression model with an adjusted odds ratio (aOR) and a 95% confidence interval (CI) was used to identify the factors associated with poor glycemic control. Statistical significance was set at *p* < 0.05.

**Result:**

A total of 231 children and adolescents with type 1 diabetes mellitus were included. The magnitude of poor glycemic control was 166 (71.9%) with 95% CI 66.0–77.7%). In multivariable analysis, the age of the child (aOR = 0.19, 95% CI: 0.05–0.83), education of the caregiver (aOR = 4.13;95% CI: 1.82–9.46), meal frequency less than three (aOR = 3.28; 95% CI: 1.25–8.62), and consumption of forbidden foods (aOR = 3.17; 95% CI: 1.21–8.29) were factors significantly associated with poor glycemic control.

**Conclusion:**

Two-thirds of participants had poor glycemic control. There was a statistically significant association between the age of the child, education of the caregiver, meal frequency, and forbidden foods with poor glycemic control. To improve glycemic control, diabetes education on meal use and selection should be conducted during follow-up along with parent education.

## Background

Diabetes mellitus (DM) is a cause of public health crisis among noncommunicable diseases that is associated with a significant increase in morbidity and mortality among the global population [1]. Worldwide, more than 500 000 children under the age of 18 years are affected by type one diabetes mellitus (T1DM), with an average annual incidence of 3 to 4% [1, 2]. The incidence of pediatric DM varies from 4.4 per 100,000 to 20 per 100,000 children in African countries [3]. Although there are different categories of DM, T1DM is the most prevalent in the pediatric population [4]. T1DM is a severe and long-lasting progressive condition that develops when the pancreas produces insufficient amounts of insulin [5].

Acute metabolic abnormalities such as diabetic ketoacidosis, hypoglycemia, and chronic consequences of diabetes, such as retinopathy, nephropathy, neuropathy, ischemic heart disease, and arterial blockage with gangrene of the extremities, are considered to be high contributors to morbidity and mortality among diabetic children [6]. The most powerful modifiable predictor for the occurrence of both acute and chronic complications of diabetes is poor glycemic control. Glycemic control is a watchword given for the level of blood sugar in the diabetic population, and strict glycemic control can postpone the onset, decrease the progression of chronic diabetes complications and increase cognitive functioning, as supported by the diabetes control and complications trial [4].

According to a study conducted in low and middle-income countries (LMICs), the magnitude of poor glycemic control ranges from 72–90.2% [7–10]. which implies that children and adolescents with T1DM have poor glycemic control. This in turn leads to deprived health outcomes [11]. In LMICs, the prognosis of children with T1DM is miserable, and many children lack timely diagnosis, treatment, and monitoring which drastically shortens life expectancy to less than one year [4, 11].

Available evidence indicates that children with T1DM have different risk factors for poor glycemic control. For example, glycosylated hemoglobin (HbA1c) levels in children are influenced by many factors, such as age, socioeconomic status, and family structure [8, 12]. It also correlated with diabetes-related characteristics, such as diabetes duration, adherence, and caregiver involvement in the child's treatment. Furthermore, the frequency of self-monitoring of blood glucose, food, duration of diabetes, frequency of clinic visits, use of an insulin regimen, and family engagement in diabetes-related activities are linked to the patient's level of glycemic control [11]. However, the abovementioned factors have been identified in high and middle-income countries, but it is unknown whether these or other variables govern glucose management in an environment where very limited access to medical care is frequently observed [11, 13, 14]. In addition, evidence on the current status of glycemic control among children with diabetes in resource-limited settings has not been updated, and the available data were based on fasting blood sugar levels alone.

To reduce the burden of poor glycemic control, various guidelines and recommendations have been made, such as those from the American Diabetes Association (ADA), the international society of pediatrics and adolescent diabetes (ISPAD), and the noncommunicable disease management guidelines of the Ministry of Health (MoH) Ethiopia [15–17]. Pediatric T1DM is always managed with aggressive insulin regimens, whether by several daily injections or continuous subcutaneous insulin infusions [18]. For all pediatric patients with diabetes, the ISPAD recommends routine self-monitoring of glucose using precise finger stick blood glucose measurements, with or without continuous glucose monitoring or intermittently scanned along with hemoglobin A1C (HgA1c) analysis every 2–3 months. Therefore, regular blood glucose monitoring should be advocated in T1DM children and adolescents to improve metabolic management. Moreover, providing support to families with children with diabetes, such as medical, financial, and social assistance, is crucial for effective diabetes management and control [1].

However, due to multiple reasons, such as parental illiteracy, poor healthcare infrastructure, and limited resources, children with diabetes suffer from poor glycemic control that persists across low-income countries, including Ethiopia. In addition, there is a paucity of data on the magnitude of poor glycemic control and its predisposing factors in children and adolescents in Ethiopia, particularly in Harari hospitals. Therefore, this study aimed to assess the magnitude of poor glycemic control and identify the associated factors among T1DM children on followed up at DM clinics in Harar eastern Ethiopia.

## Methods and materials

### Study setting, design, and population

This multicenter, cross-sectional study was conducted at public hospitals in the Harari region, eastern Ethiopia. Hiwot Fana Comprehensive Specialized University Hospital is a teaching referral hospital at Haramaya University and Jugol General Hospital located in Harar Town. These hospitals serve more than six million people in eastern Ethiopia. All T1DM children and adolescents who were followed up at a Hiwot Fana Compressive Specialized University Hospital and Jugol General Hospital from November 15, 2022 to January 15 2023 were included. Children aged less than or equal to 18 years and who were diagnosed with T1DM with regular follow-up were involved.

### Inclusion and exclusion criteria

Children and adolescents with at least one HbA1c level and/or three consecutive measurements of fasting blood sugar (FBS) between November 15, 2022 to January 15 2023 were included. However, children and adolescents who were on treatment for less than 3 months were not included in this study.

### Sample size and procedure

The sample size was determined by a single proportion formula by considering 16.4% glycemic control as a proportion (P) from a previous study conducted in Ethiopia [4] with the following assumptions 95% confidence interval (CL) and marginal error of 5%. The calculated sample size was 210; we added a 10% nonresponse rate, and the final sample size was 231. The sample was proportionally allocated to the selected hospitals based on the estimated average follow-up in the previous three months (January to March 2022) in both hospitals. A total of 231 participants were included using a consecutive sampling technique from those who were on follow-up with T1DM from November 15, 2022 to January 15, 2023, and who fulfilled the inclusion criteria.

### Data collection

Data were collected using a pretested, structured interviewer-administered questionnaire and a review of medical records with a validated data abstraction checklist adapted from previous studies [4, 11–14]. The tool contains information on the sociodemographic conditions of the caregiver and children such as age, sex, educational status, marital status of caregiver, occupational status, and place of residence); clinical characteristics and medication-related factors (duration of diabetes, medication duration, anthropometric parameters, insulin type, and amount, follow-up, comorbidities, and medication use and place of insulin storage); nutritional-related factors (the number of meals per day, meal content, consuming forbidden foods); and health facility related factors (frequency of clinic visits, a distance of health facility, means of transportation, counseling by a health professional, presence of health insurance). HbA1c and FBG levels were measured according to the hospital standard of practice for laboratory procedures. HbA1c was measured using high-performance liquid chromatography, and a level greater than 7.5% indicated poor glycemic control [1].

Data were collected by two BSc nurses supervised by two senior nurses with a second degree. Patient records were retrieved using the medical registration number identified in the total DM caseload in the registration follow-up logbook. Data collection was carried out over two months, from November 15, 2022 to January 15, 2023.

#### Poor glycemic control

 Is defined as HbA1c is more than 7.5% and/or the average FBG level is either < 70 or > 145 mg/dl [1].

#### Good glycemic control

Is defined as HbA1c is less than 7.5% and/or the average FBG level is either > 70 or < 145 mg/dl [1].

#### Length

Measured with the sliding board if age was less than 2 years and more than that, was measured by standing board and older measured meter.

#### Weight

Measured with a weight scale with calibration for each participant and interpreted as follows: Weight for age, between 3 and 2SD – normal and below 2SD- malnourished; Length/height for age, between 3 and 2SD – normal and below 2SD- malnourished; Weight for Length/height, between 3 and 2SD – normal and below 2SD- malnourished; Body Mass Index (BMI) for age, between 3 and 2SD – normal and below 2SD- malnourished.

#### Consuming forbidden foods

Is defined as if children consumed bread, pasta, rice, and sugar-added foods on a regular or daily basis.

### Data quality control

To ensure the quality of data, a pretest was conducted on 5% of participants at Dil Chora referral hospital of the DM follow-up clinic. Two days of training were provided to all data collectors and supervisors. The data collection process was closely supervised, and the completeness of each questionnaire was checked daily by supervisors and the principal investigator. During data cleaning, a logical checking technique was employed to identify errors. Finally, double data entry was performed to verify the data consistency.

### Data processing and analysis

The collected data were checked for completeness and coded, entered into Epi Data version 4.6, and exported to SPSS 26 for analysis. Frequency, means and proportions were used for the descriptive analysis. Glycemic control was classified as good if FBG was between 70–145 mg/dl or HbA1c level was < 7.5% and out of these values indicated poor glycemic control. A binary logistic regression model was used to determine the association between each independent variable and the outcome variable. All variables with *p* ≤ 0.25 in the bivariable logistic regression were entered into the final multivariable analysis to control confounders. The goodness of fit of the model was tested using the Hosmer‒Lemeshow test (> 0.05). A multicollinearity test was performed to determine the correlation between the independent variables using variance inflation factors (VIF > 10). The adjusted odds ratio (aOR) with 95% confidence intervals (CI) and a *p value* less than 0.05 were considered a statistically significant association.

## Results

### Sociodemographic characteristics of the participants

**Fig. 1:**
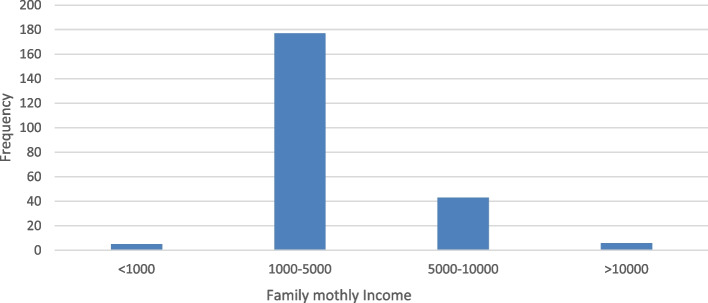
Monthly family income of children and adolescents with T1DM in Jugol General Hospital and Hiwot Fana Compressive Specialized University Hospital, Harar, Eastern Ethiopia, November 15, 2022 to January 15, 2023. (*n* = 231)

Among 231 study participants included in this study, 129 (55.8%) were male. The age of the patients ranged from 1–18 years, with a mean age of 13 ± 4.9 years, and those between the ages 10 and 18 accounted for 124 (53.7%). Regarding educational status, two third of the participants 155 (67.1%) were in elementary school and 57 (24.7%) were in high school. Of the study participants, 144 (62.3%) were from rural areas. The average standard deviation (SD) of family size was 6 ± 2.1 and approximately three-quarters of the participants had a family size ranging from 5 to 10. The mean and SD of the family's monthly income were 4379 ± 4904.7 ETB and 177 (76.6%) of them earned between 1000 and 5000 birrs (Fig. 1, Table 1).

**Table 1:** Sociodemographic characteristics of children and adolescents with T1DM in Jugol General Hospital and Hiwot Fana Compressive Specialized University Hospital, Harar, Eastern Ethiopia, November 15, 2022 to January 15, 2023. (*n* = 231)

Variables	Category	Frequency	Percent
Sex	Male	129	55.8
	Female	102	44.2
Age	Preschool	21	9.1
	School-age	86	37.2
	Adolescent	124	53.7
Place of residence	Urban	87	37.7
	Rural	144	62.3
Educational status	Preschool	19	8.2
	Elementary	155	67.1
	Secondary	57	24.7
Family size	2–4	57	24.7
	5–10	167	72.3
	> 10	7	3

### Parental sociodemographic characteristics

Fathers were caregivers for 72 (28.6%) of the patients, 66 (31.2%) were cared by mothers and 54 (23.4%) of the participants had no caregivers. Of the 177 caregivers in the study, 91 (51.4%) were female and 86 (48.6%) were male. The age of the caregivers ranged from 18 to 75 years, with a mean of 36.8 ± 10.18 years. Half of the caregivers 89 (50.2%) were unable to read and write and only 43 (24.7%) attended secondary school or above. More than three-fourths of the caregivers, 139 (78.5%) were married and 27 (15.3%) were single. Seventy-nine of the caregivers (44.6%) were farmers, and 34 (19.2%) were housewives (Table 2).

**Table 2:** Sociodemographic characteristics of caregivers of children and adolescents with T1DM in Jugol general hospital and Hiwot Fana Compressive Specialized University Hospital, Harar, Eastern Ethiopia, November 15, 2022 to January 15, 2023. (*n* = 231)

Variables	Category	Frequency	Percent
Primary caregiver	Mother	66	28.6
	Father	72	31.2
	Sister/brother	13	5.6
	Others	26	11.3
	No caregiver	54	23.4
Sex	Male	86	48.6
	Female	91	51.4
Age	18–25	16	9
	26–35	73	41.2
	36–45	59	33.3
	46–55	24	13.6
	56–65	4	2.3
	66–75	1	0.6
Educational level	Unable to read and right	89	50.2
	Primary	45	25.4
	Secondary	43	24.7
Marital status	Single	27	15.3
	Married	139	78.5
	Separated	2	1.1
	Divorced	4	2.3
	Widowed	5	2.8

### Nutritional characteristics

Of the total participants, 109 (47.2%) had three meals and one snack per day. Among the participants, 50 (21.6%) had consumed forbidden food, and two-thirds of them 35 (70%) consumed foods that had a high content such as bread, pasta, and macaroni. Of the participants, 45 (19.4%) were underweight, 62 (21%) were stunted, 60 (25.9%) were wasting, 55 (23.8%), and 22 (9.4%) were malnourished when assessed with WFA, L/HFA, W/H, BMI for age and MUAC respectively (Table 3).

**Table 3:** Nutritional characteristics of children and adolescents with T1DM in Jugol General Hospital and Hiwot Fana Compressive Specialized University Hospital, Harar, Eastern Ethiopia, November 15, 2022 to January 15, 2023. (*n* = 231)

Variables	Category	Frequency	Percent
Meals	3 meals and 2 snacks	47	20.3
	3 meals and 1 snack	109	47.2
	3 meals only	37	16
	Less than 3 meals	38	16.5
Meal content	Carbohydrate	8	3.5
	Protein	7	3
	Vitamins	2	0.9
	Fiber	2	0.9
	All	212	91.8
Forbidden food	Yes	50	21.6
	No	181	78.4
Type of forbidden	Foods sugar is added	15	30
Food	bread, Pasta, and macaroni	35	70
WFA	Sever Underweight	45	19.4
	Moderate underweight	15	6.4
	Normal	171	74.1
H/LFA	Sever Stunted	26	8
	Moderately stunted	36	13
	Normal	169	79
WFH	Malnourished	60	25.9
	Well-nourished	171	74.1
BMI for age	Malnourished	55	23.8
	Well-nourished	155	67
	Not assessed	21	9
MUAC	Malnourished	22	9.4
	Well-nourished	209	90

*WFA* Weight for age, *H/LFA* height or length for age, *WFH* weight for height, *BMI* body mass index, *MUAC* middle upper arm circumference

### Duration and clinical characteristics

More than half (58.4%) of the study participants’ mean age at diagnosis was 7–12 years with SD of 9 ± 4.2 and duration of doagnosis range and SD of 1–5, and 3.6 ± 2.7 years respectively. This implies that the patients were on medication for a mean SD of 3.6 ± 2.6 years. The majority of the study participants, 213 (92.2%) were on nonpremixed NPH/RI regimen, while 6 (2.6%) used premixed NPH/RI insulin. The mean dose of insulin is 0.85 ± 0.24 IU/kg and 109 (47.2%) of the patients use a refrigerator to store the insulin. One handred seventy six (76.2%) of the study participants have administered the insulin by themselves and only 21 (9.1%) missed one dose of insulin. Three-quarters of the patients (75.3%) had their glucometer and approximately three-fourths of them (78%) used it 3 or more times a week. Only 13 (5.6%) of the study participants had comorbidities, with epilepsy accounting for 6 (46.2%). All injections that were administered and supervised in the last 24 h by the caregiver accounted for 61 (26.4%) and 71(30.7%) of the cases respectively. Seventy-five (42.4%) of the caregivers set up the meter, performed the finger prick, or supervised the task, while 70 (39.5%) had no participation in all care activities. Almost all of the patients 223 (96.5%) did not use any medications that could increase blood sugar (Table 4).

**Table 4:** Clinical characteristics of children and adolescents with T1DM in Jugol General Hospital and Hiwot Fana Compressive Specialized University Hospital, Harar, Eastern Ethiopia, November 15, 2022 to January 15, 2023. (*n* = 231)

Variables	Category	Frequency	Percent
Age at Diagnosis (years)	1- 3	18	7.8
	4- 6	35	15.2
	7- 12	135	58.4
	13- 18	43	18.6
Duration of DM(years)	< 1	55	23.8
	1- 5	119	51.5
	> 5	57	24.7
Duration of medications(years)	< 1	56	24.2
	1- 5	117	50.6
	> 5	58	25.1
Insulin regimen	Premixed NPH/RI	6	2.6
	Nonpremixed NPH/RI	213	92.2
	NPH alone	12	5.2
Insulin dosage	< 0.8 IU/Kg	84	36.4
	0.8–1.2 IU/Kg	119	51.5
	> 1.2 IU/Kg	28	12.1
Storage	Refrigerator	109	47.2
	Pot	50	21.6
	other	72	31.2
Who administers	Child himself	174	75.3
	Caregivers	54	23.4
	A child with the support of a caregiver	3	1.3
Missed dose	No missed dose	210	90.9
	1 missed dose	21	9.1
Glucometer	yes	174	75.3
	No	57	24.7
Frequency of BGM	≥ 3 × weekly	156	67.5
	1–2 × weekly	18	7.8
	Non-per week	17	24.7
Co-morbidities	Yes	13	5.6
	No	218	94.4
Type of co-morbidity	Epilepsy	6	2.6
	Cardiac diseases	1	4
	HIV/RVI	2	0.9
	Others	4	1.7
	None	218	94.4
Dose injected by a caregiver	None	111	62.7
	1 injection	5	2.8
	All injections	61	34.5
Dose supervised by a caregiver	None	104	58.8
	1 injection	2	1.1
	All injections	71	40.1
Participation of caregivers	No participation	70	39.5
	Reminds the child	32	18.1
	Sets up the meter	75	42.4
Medications that increase blood glucose	Yes	8	3.5
	No	223	96.5

*DM* diabetes Mellitus, *BGM* blood glucose measurement, *NPH/RI* neutral protamine Hagedorn/regular insulin, *IU/kg* international unit per kilogram, *HIV/RVI* human immune deficiency virus/retroviral infection

### Health system characteristics

The average distance of study participants’ residency from health facilities was 72 ± 86 km, and 188 (81.4%) used public transport as a means of transportation. The average time taken from home to the health facility was 2 to 3h. Almost all of the patients 229 (99.1%) received counseling during the diagnosis of diabetes and 201 (87%) received all of the advice related to diabetes and insulin. A total of 211 (91.3%) patients had follow-up every month, while 20 (8.7 %7%) patients had follow-up every 2 months. Forty (17.3%) patients said that they faced problems in the hospital during health care delivery, with long waiting times, accounting for 27 (67.5%). Third- fourths of the study participants 174 (75.3%) had health insurance (Table 5).

**Table 5:** Health system characteristics of children and adolescents with T1DM in Hiwot Fana Compressive Specialized University Hospital and Jugol General Hospital, Harar, Eastern Ethiopia, November 15, 2022 to January 15, 2023. (*n* = 231)

variables	category	Frequency	Percent
Distance in Kilometer (Km)	< 10	70	30.3
	10–49	51	22.1
	50–99	45	19.5
	100–199	34	14.7
	200–299	8	3.5
	≥ 300	2	0.9
	Not known	21	9.1
Transportation	On foot	43	18.6
	Public transport	188	81.4
Time for arrival	< 1 h	146	63.2
	1 h and more	85	38.6
Counseling	Yes	229	99.1
	No	2	0.9
Types of advice	Importance of insulin	6	2.6
	Method of insulin use and glucometer	13	5.6
	Complications of diabetes	4	1.7
	Not to miss doses and appointments	5	2.2
	Other	2	0.9
	All	201	87
Follow-up frequency	1 month	211	91.3
	2 months	20	8.7
Problems in the hospital	Yes	40	17.3
	No	191	82.7
What problems	Shortage of insulin	7	3
	Long waiting time	27	11.7
	Poor communications	6	2.6
	None	191	82.7
Insurance	Yes	174	75.3
	No	57	24.7

### The magnitude of poor glycemic control

**Fig. 2:**
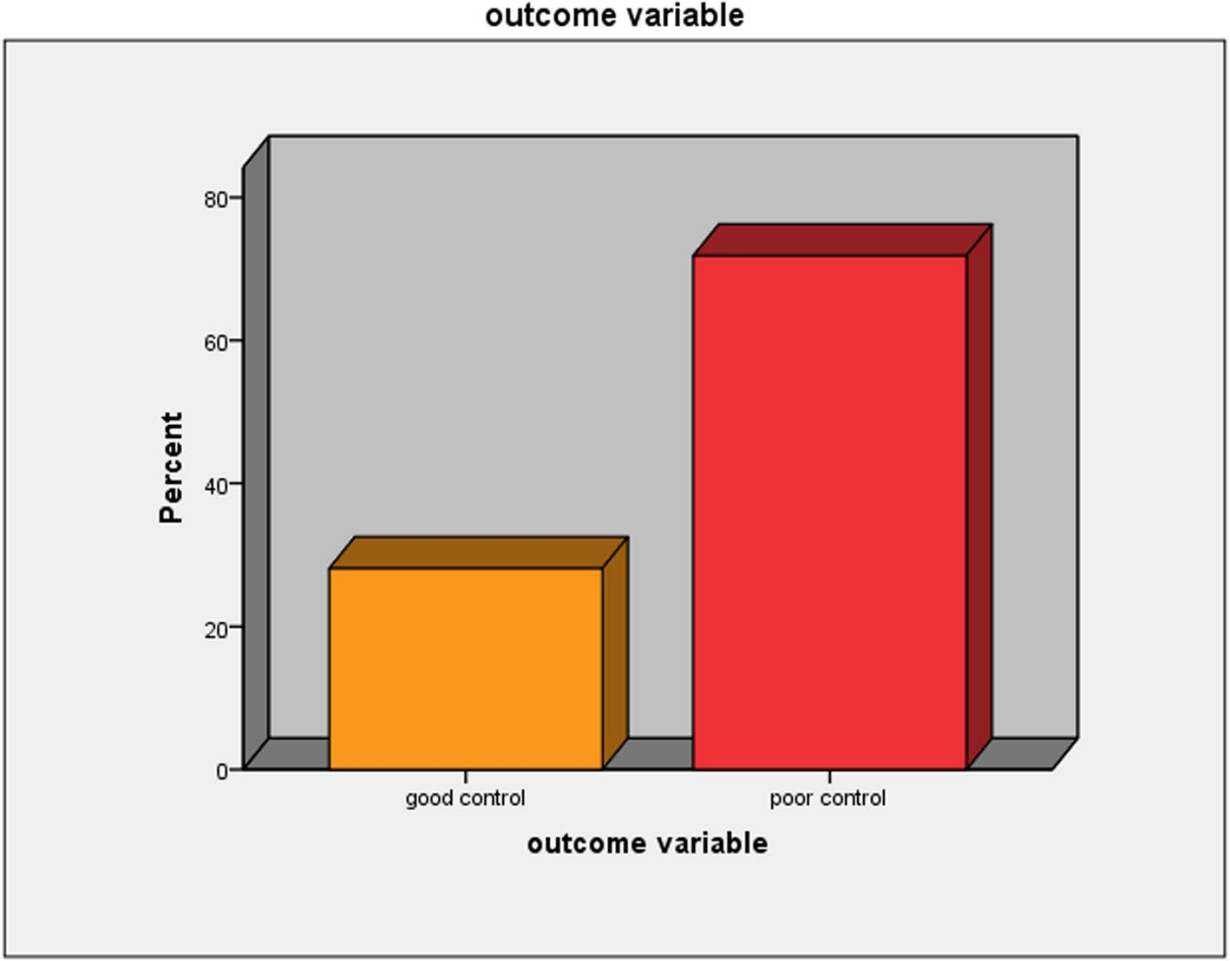
Glycemic control of children and adolescents with T1DM in Jugol General Hospital and Hiwot Fana Compressive Specialized University Hospital, Harar, Eastern Ethiopia, November 15, 2022 to January 15, 2023. (*n* = 231)

Of the total participants, more than two-thirds had poor glycemic control (71.9%) (95% CI: 66.0–77.7%). The mean and median HgbA1c levels were 10.4 and 10.8% respectively. The mean and median FBG levels were 220 and 200 mg/dl respectively. The mean HgbA1c and FBG were 10.4 ± 3.1 and 220 ± 116 respectively (Fig. 2).

### Factors associated with poor glycemic control

In the bivariable logistic regression analysis, the age of the child, education level of both the child and caregiver, meal frequency, glucometer, BMI of the study participants who administered the medication, and consumption of forbidden food were eligible for the final model. However, only the age of the children, educational level of the caregiver, consumption of forbidden foods, and meal frequency were significantly associated with poor glycemic control in children and adolescents with T1DM.

Accordingly, preschool children were less likely by 81% to have poor glycemic control than those aged between 10 and 18 years (aOR = 0.19; 95% CL: 0.05–0.83). Those children who had a caregiver who was unable to read and write were four times more likely to have poor glycemic control than those who had secondary education (aOR = 4.13;95% CI: 1.82–9.46). Moreover, children whose meal frequency less than three were 3.28 times more likely to develop poor glycemic control than those children with a meal frequency of three and more than two snacks (aOR 3.28; 95% CI: 1.25–8.62). Furthermore, patients who ate forbidden foods were 3.17 times more likely to have poor glycemic control than their counterparts (aOR 3.17; 95%CI: 1.21–8.29) (Table 6).

**Table 6:** Factors associated with poor glycemic control among children and adolescents with T1DM in Jugol General Hospital and Hiwot Fana Compressive Specialized University Hospital, Harar, Eastern Ethiopia, November 15, 2022 to January 15, 2023. (*n* = 231)

Variables	Poor Control	Good control	COR(95%CI)	AOR (95%CI)	*P* value
Age of a child					
Preschool	10	11	0.30(CI 0.12–0.78)	0.19(CI 0.05–0.83)	0.027^*^
School-age	63	23	0.91(CI 0.49–1.71)	1.42(CI 0.61–3.32)	0.414
Adolescent	93	31	1		
Education of a child					
Preschool	10	9	0.47(CI 0.16–1.37)	0.64(CI 0.16–2.58)	0.530
Primary	116	39	1.26(CI 0.65–2.48)	0.64(CI 0.27–1.50)	0.304
Secondary	40	17	1		
Education of caregiver					
Cannot read and write	96	20	4.00(CI 1.98–8.19)	**4.13 (CI 1.82–9.46)**	0.001^*^
Primary	39	19	1.72(CI 0.80–3.67)	1.78(CI 0.76–4.17)	0.183
Secondary	31	26	1		
Meal frequency					
Less than 3 meal	41	7	2.92(CI 0.22–7.02)	**3.28 (CI 1.25–8.62)**	0.016^*^
3meal only	31	11	1.4(CI 0.65–3.05)	1.62(CI 0.68–3.86)	0.280
3meal and more than 2 snacks	96	47	1		
Glucometer					
Yes	130	44	1.72(CI 0.91–3.26)	1.77 (CI 0.84–3.71)	0.131
No	36	21	1		
BMI					
Malnourished	46	9	2.39(CI 1.09–5.212)	2.37(CI 0.91–6.15)	0.076
Well-nourished	120	56	1		
Who administers the medication					
Child himself	130	44	1.72(CI 0.91–3.26)	0.90(CI 0.34–2.40)	0.830
Caregiver	36	21	1		
Consumption of forbidden food					
Yes	43	7	2.89(CI 1.23–6.83)	**3.17(CI 1.21–8.29)**	0.019^*^
No	123	58	1		

*COR *crude odds ratio, *AOR *adjusted odds ratio, *BMI *body mass index

^*^indicates statistically significant variable with multivariable logistic regression

## Discussion

This study aimed to assess the magnitude of poor glycemic control and its associated factors among children and adolescents with T1DM in the Harari region, eastern Ethiopia. Accordingly, the magnitude of poor glycemic control was 71.9% (95% CI: 66.0–77.7%). In this study, the age of the patients, education of the primary caregiver, meal frequency, and consumption of forbidden food were independently associated with poor glycemic control.

The findings of this study are almost comparable with those reported in Arabian Gulf countries, African adolescents, and Sudanese children 72.6%, 67.4%, and 76% had poor glycemic control respectively [7, 19, 20]. However, the present findings are relatively lower than those of studies conducted in southern Ethiopia (83.6%) [4], Jimma (84%) [21], Sudan (76–78.9%) [7, 22] and Tanzania (97.3%) [9, 23]. This discrepancy could be explained by the vast differences in population sizes employed in the study. It may also be due to differences in the quality of care delivered to the patients as of time run in all of the study area. On the other hand, the findings in this study are much higher than the study conducted in the northwest part of Ethiopia (39.3%) [24]. This reveals how crucial it is to reduce our population's HbA1c level within an optimal range to minimize the devastating events that can occur following a poor level of glycemia among diabetic children in this particular study setting.

Glycemic control in children is influenced by a wide range of factors, such as young age, sex, BMI, socioeconomic status, education level, duration of diagnosis, and primary caregiver [11, 14, 25]. In this study, preschool-aged children were less likely by 81% to have poor glycemic control than adolescents. This finding is consistent with studies conducted in Dar es Salaam Tanzania and Egypt [9, 10, 12, 13]. The reason can be associated with parental participation and supervision in this age group [26, 27]. This is also because as a child matures, they undergo a variety of physical and lifestyle alterations [28, 29]. Furthermore, this might be due to hormonal effects and a decline in parental supervision of different clinical aspects of diabetes care in adolescents [14, 16].

Education of the primary caregiver which is linked to glycemic control is an independent factor. Children with parents who cannot read and write have poor glycemic control and a four-fold increased risk of poor glycemic control compared to children supported by educated parents. This is in agreement with studies conducted in Tanzania and Bulgaria, which reported that children and adolescents from families with poor parental education frequently struggle to achieve and maintain effective diabetes management [23, 30]. This is due to its effect on the child's care, follow-up, and correct drug handling and delivery [8, 26, 27].

In this study, patients who consumed foods with high levels of carbohydrates and sugar were 3.2 times more likely to have poor glycemic control than those who did not consume these foods. This finding is consistent with research conducted in Germany, Austria, and Italy, which signifies a link between consuming fewer carbohydrates, totally restraining food containing much darling, and lower HbA1c readings [25, 31–33]. These results suggest the need for medical nutrition therapy and attention to diabetes education to help children overcome barriers that impair glycemic control [34]. This finding also implies that monitoring carbohydrate intake, whether by carbohydrate counting or experience-based estimation is the key to achieving glycemic control [35]. An international organization such as the ISPAD recommends further research to examine any potential metabolic and glycemic benefits of carbohydrate restriction in the treatment of DM [36].

Frequency of meal was another independent factor significantly associated with poor glycemic control. A meal frequency of less than three were 3.3 times more likely to have poor glycemic control as compared to their counterparts having a meal frequency of three and more than two snacks. The findings of this study are consistent with research carried out in Japan, which stated that those with low meal frequency increases the mean 24 h interstitial glucose concentration among young diabetic population [37]; this finding also supported by the study done in Germany [38] and USA [39, 40]. The frequency of diet among T1DM may differ from that of healthy individuals due to disease-related factors that may affect the course of diabetes; waiting too long between meals and/or eating one big meal can rise blood sugar level [37]; furthermore, skiping meal frequently can lower metabolism, making it actually harder to manage blood glucose [41]. This insight will have an important implication in determining mealing approach to individuals with diabetes; recent evidence suggests that both meal frequency and daily energy distribution can influence glycaemic control [41, 42]. Similarly,in studies of meal frequency, conducted in individuals with T1DM, consumption of breakfast, the habit of regular meal pattern with multiple smaller meals (4–7 meals per day) and more frequent meals have been associated with better glycaemic control [41, 43].

In general, this study points to positive implications for clinical care, health service management, and research within the area of diabetic specialization. Clinically, healthcare workers can help patients by focusing on identifed factors associated with glycemic control among T1DM children at clinical setting. Healthcare managers can access current evidence regarding the overall magnitude of glycemic control in this particular study setting to take remedial action to strengthen service delivery by healthcare providers and other stakeholders. Researchers can also be motivated to conduct further research in this area emphasizing on diabetic nutritin by taking this study as a preliminary finding.

## Limitations of the study

The study might be prone to recall bias for some intellectual questions and FBG measurements obtained from medical records might be subject to measurement errors that lead to underestimation or overestimation of the result. However, an effort was made to overcome these issues by taking the mean value of three-month consecutive values of FBG measurements and HA1c values in almost all patients.

## Conclusion

Two-thirds of the participants had poor glycemic control. There was a statistically significant association between the age of the child, education of the caregiver, meal frequency, forbidden foods with poor glycemic control. To improve glycemic control, diabetes education including meal utilization, selection, and integrating the finding into routine care will help to address gaps in caregiver nutritional literacy and their ability to provide appropriate care.

## Data Availability

Data is available upon reasonable request from the corresponding author.

## Abbreviations

ADA: American Diabetes Association
AOR: Adjusted odd Ratio
BMI: Body Mass Index
DM: Diabetes Mellitus
FBG: Fasting Blood Glucose
HbA1c: Glycated Hemoglobin A1C
IHREC: Institutional Health Research Ethical Committee
ISPAD: International Society of Pediatrics and Adolescent Diabetes
SD: Standard Deviation
SPSS: Statistical Package for Social Sciences
T1DM: Type 1 Diabetes Mellitus
